# “Growth patterns in children with mucopolysaccharidosis type I-Hurler after hematopoietic stem cell transplantation: Comparison with untreated patients”

**DOI:** 10.1016/j.ymgmr.2021.100787

**Published:** 2021-08-09

**Authors:** Alessandro Cattoni, Sofia Chiaraluce, Serena Gasperini, Silvia Molinari, Andrea Biondi, Attilio Rovelli, Rossella Parini

**Affiliations:** a Department of Pediatrics, Università degli Studi di Milano Bicocca, Fondazione Monza e Brianza per il Bambino e la sua Mamma, Azienda Ospedaliera San Gerardo, Monza, (MB), Italy; b TIGET Institute, IRCCS San Raffaele Hospital, Segrate, (MI), Italy

**Keywords:** Mucopolysaccharidosis I, Hurler disease, Hematopoietic stem cell transplantation, Growth, HSCT, hematopoietic stem cell transplantation, MPS-IH, mucopolysaccharidosis I Hurler, SDS, standard deviation score, GAGs, glycosaminoglycans, ERT, Enzyme replacement therapy., WHO, World Health Organization, MPH, midparental height, EBMT, European Blood and Marrow Transplantation Society, IDUA, alpha-L-iduronidase

## Abstract

The impact of hematopoietic stem cell transplantation (HSCT) on growth in patients diagnosed with mucopolysaccharidosis I Hurler (MPS-IH) has been historically regarded as unsatisfactory. Nevertheless, the growth patterns recorded in transplanted patients have always been compared to those of healthy children.

The objective of this study was to verify the impact of HSCT on MPS-IH long term growth achievements. The auxological data of 15 patients were assessed longitudinally and compared both to the WHO growth centiles for healthy individuals and to recently published curves of untreated MPS-IH children. Despite a progressive decrease after HSCT when estimated with reference to the WHO growth charts, median height SDS showed a progressive and statistically significant increase when comparing the stature recorded at each timepoint in our population to the curves of untreated MPS-IH individuals (from ‐0.39 SDS at t_0_ to +1.35 SDS 5 years after HSCT, *p value <* *0.001* and to +3.67 SDS at the age of 9 years, *p value <* *0.0001*).

In conclusion, though not efficient enough to restore a normal growth pattern in MPS-IH patients, we hereby demonstrate that HSCT positively affects growth and provides transplanted patients with a remarkable height gain compared to untreated gender- and age- matched individuals.

## 1 Introduction

The mucopolysaccharidoses (MPS) are a heterogeneous group of inborn errors of metabolism arising from the defective activity of enzymes involved in the degradation of mucopolysaccharides (or glycosaminoglycans, GAGs). As a result, the pathological lysosomal storage of heparan sulfate, dermatan sulfate, chondroitin sulphate and/or keratan sulfate in several tissues potentially leads to multi-systemic complications, such as abnormal facial features, cardiac defects, hepatosplenomegaly, upper airways obstruction, corneal clouding, skeletal dysplasia and progressive central nervous system deterioration [1].

In addition, faltering growth is a frequent finding in patients with MPS, often resulting in overt short stature. Though still not completely understood, the pathogenesis of growth impairment in patients with MPS can be mostly regarded as the effect of the pathological storage of GAGs in cartilages, bone and growth plate, resulting in inflammation, apoptosis and poorly organized connective tissue matrix [2]. Furthermore, it has been demonstrated in murine models that the accumulation of GAGs plays a detrimental effect on bone deposition by inducing a dysfunctional osteoblastic activity. Conversely, osteoclastogenesis is remarkably increased, involving an imbalance between ineffective osteoblastic bone deposition and triggered osteoclast-driven bone reabsorption [3].

A detrimental combination of growth plates dysfunction, augmented bone reabsorption and the stigmata of multiple dysostosis (*i.e.* kyphosis, scoliosis, genu valgum) severely affects the final height attained [4].

The growth patterns for untreated patients diagnosed with several types of mucopolysaccharidoses have been systematically described [5,6]. Though the timing of progressive growth deceleration varies among different types of mucopolysaccharidoses, short stature upon final height attainment is a common outcome [5]. The clinical spectrum ranges, in terms of final height achieved, from Hurler syndrome and MPS IV, as the most severely affected forms, to MPS III, that shows the mildest growth impairment.

Enzyme replacement therapy (ERT) and hematopoietic stem cell transplantation (HSCT) are the standard-of-care in patients with MPS, as they remarkably improve patient's life expectancy and quality by progressively depleting GAGs storages [[7], [8], [9]]. Nevertheless, the literature provides conflicting data about the impact of ERT and HSCT on musculoskeletal disease and overall height gain [4,10].

The effect of ERT on growth has been assessed in MPS I [11], MPS II [6,12], MPS IV [13] and MPS VI [14]. Though some Authors reported a potential height gain after ERT (MPS I, MPS II and MPS VI), these results were very limited, unless enzyme therapy was undertaken during the first weeks of life. In addition, other reports demonstrated no improvements at all (MPS IV).

Despite positive and superimposable effects of ERT and HSCT on growth in an analysis held by Patel and colleagues in MPS II patients [6], the role of transplantation on height gain has been widely debated in patients with different MPS sub-types, with most Authors agreeing on an overall poor response [[15], [16], [17]].

This seems to be related to the widely described detrimental effects of the pathologic accumulation of GAGs in the bones and growth plates [18,19], likely related in part to the avascular nature of growth plate cartilage making this tissue less accessible to therapeutic agents. Indeed, several Authors highlighted that the poor correction of short stature in MPS patients receiving HSCT could be related to its inability to normalize endochondral ossification (typically affected in MPS) due to poor cell engraftment and enzyme access to cartilage of the growth plates [9,16,18].

HSCT, being the only available treatment able to cross the blood brain barrier, is the gold standard of treatment for MPS I Hurler (MPS-IH, OMIM #607014), the most severe form of MPS I, where developmental delay starts early and progresses rapidly in absence of treatments [20]. Hundreds of patients with MPS-IH have been transplanted so far and some data on growth after HSCT have been reported in the literature. Staba and colleagues documented normal growth velocities during a brief follow-up with a maximum of 6 years after HSCT [21].

On the other hand, longer-term data on growth in transplanted MPS-IH showed progressive deviation from the reference curves, with a large proportion of patients presenting with a final height below −2 SDS [4,17,22,23].

However, MPS-IH growth curves after successful HSCT have never been compared to curves of untreated patients. Viskochil and colleagues have recently published the growth curves of untreated MPS-I patients, using data obtained from the MPS-I Registry [24]. Disease-specific growth charts were constructed on the basis of the auxological data available for 463 untreated individuals affected with MPS-IH.

The aim of the present analysis was to retrospectively assess the growth patterns in MPS-IH patients treated with HSCT in our Institution, by providing a systematic comparison with the reference auxological data of healthy children and with those recently published for untreated MPS-IH individuals.

## 2 Materials and metods

We performed a retrospective, observational, monocentric study.

### 2.1 Patients' enrollment criteria

The study cohort included all those patients diagnosed with MPS-IH and subsequently treated with HSCT at our Institution between 1st January 2000 and 31st December 2015. All the patients had been followed-up for at least 60 months after transplantation from a clinical and auxological perspective.

Conditions that led to patients' exclusion were: total body irradiation (TBI) as a conditioning schedule administered before HSCT due to its independent detrimental effect on growth, treatment with recombinant human growth hormone or evidence of growth hormone deficiency, incomplete auxological data available before and after HSCT.

From an endocrine perspective, all the patients have been reviewed six-monthly by an endocrinologist with a specific commitment on inborn errors of metabolism. Insulin-like growth factor-I (IGF-I) levels have been assessed in all the patients enrolled and none of the children showed values < −2 SDS with reference to gender- and age-specific ranges (median IGF-I SDS: −0.36, range: −1.5 to 2.3). Whenever the auxological data raised the suspicion of an impairment of growth hormone secretion, the patients underwent GH stimulation tests that ruled out growth hormone deficiency for all the children assessed and included in the present analysis. In detail, GH peaks achieved after GH stimulation tests were 11.2, 9.2 and 10.1 ng/mL for patients 1, 14 and 15 respectively, with GH deficiency being defined, in our Country, by the finding of GH peaks <8 ng/mL twice after two different GH stimulation tests performed in two separate days.

Informed consent was obtained by patients' caregivers.

### 2.2 Study design and definitions

A systematic clinical and auxological follow-up had been undertaken for all the MPS-IH patients who underwent HSCT at our Institution. All the patients had been reviewed every 3 to 6 months as a part of a post-transplantation follow-up program, at least for the first 5 years after HSCT.

We retrospectively recorded and processed the auxological data measured 6 months before HSCT (t_−__6_), at the time of HSCT (t_o_) and subsequently 12-monthly for the first 5 years after transplantation (t_12_, t_24_, t_36_, t_48_, t_60_). Variations of ±1 month at any timepoint were regarded as acceptable and recorded.

In addition, auxological data collected between 9.0 and 9.99 years of age were recorded in those patients who had already achieved this age at the time of enrollment. This timepoint (t_9yrs_) was included in order to provide a longer-lasting comparison with the published centiles for untreated MPS-IH, that include patients as old as 10 years [24]. Height values recorded at older age could only be compared with the centiles for the general population but not with affected untreated patients.

Finally, adult height (t_final_) was recorded in all those fully post-pubertal patients who presented with a height velocity < 0.5 cm/year and/or evidence of epiphyseal fusion on hand-wrist X-ray for bone age assessment.

All the additional intermediate measurements available have been recorded and plotted on the WHO and disease-specific growth charts.

In those patients who underwent HSCT twice due to a loss of engraftment after the first procedure, data and timepoints have been recorded with reference to the second transplantation.

We performed a longitudinal evaluation of the growth patterns of all the patients and provided a systematic comparison with the height references of the general pediatric population and of untreated MPS IH patients.

### 2.3 Data collection and processing

Demographic, anthropometric, hematological, biochemical and radiological data were retrospectively collected from patients' clinical records and growth charts.

The following auxological data were collected for each of the timepoints cited in the previous paragraph: height, weight, BMI, height velocity (estimated over 6 or 12 months).

In younger infants, we recorded patient's length with an infantometer: the infant was placed supine and unclothed on the board and held gently with his or her body aligned, head in a neutral position and extended left leg. In patients aged 2 years or older, standing height was measured with a Harpenden stadiometer.

In order to compare our patients with age- and gender- matched healthy children, anthropometric data (height, weight and body mass index) were expressed as standard deviation scores (SDS) with reference to World Health Organization (WHO) centiles. Also, midparental height (MPH), available for most patients, was expressed as SDS with reference to the WHO centiles. We estimated, for each patient and each timepoint, the gap between MPH-SDS and height-SDS (∆ MPH-H SDS).

In order to quantify the height gain in transplanted patients after HSCT compared to untreated ones, we also expressed all the auxological data recorded before the age of 10.0 years as SDS with reference to the centiles of untreated children published by Viskochil and colleagues [24].

Height velocity was expressed as SDS with reference to the Tanner-Whitehouse growth charts [25].

### 2.4 Statistical analysis

Mean and standard deviation values, interquartile ranges and median were used to describe the study population. Continuous variables were compared with Wilcoxon rank-sum test.

Box plots with median and interquartile ranges were used to represent height and height velocity SDS.

Linear regression model was used to assess the impact of a 12- and 24-months height gain on the 60-months overall height gain.

Multiple regression model was used to assess the potential impact of demographic and auxological factors on the 60-months overall height gain after HSCT.

Statistical analysis was performed using JMP® software by SAS.

SIEDP Growth 4.0® by Eli Lilly was used to estimate the SDS for height, weight, BMI and height velocity with reference to the WHO or Tanner-Whitehouse growth charts.

A Microsoft Excel® single-sheet calculator was used to estimate height SDS with reference to the untreated MPS-IH growth charts.

## 3 Results

### 3.1 Study population

Fifteen MPS-IH patients (4 males, 26.7%) aged 15.6 ± 5.4 years (range: 4.7–22.6) at the time of data collection were included in the study population. The demographic and clinical features of the patients enrolled are showed in Table 1. All the gene variants detected in our patients and listed in the table have been described as associated to Hurler disease. The patients were diagnosed with MPS-IH at a mean age of 11.4 ± 4.3 months (range: 4.9–18.1).

**Table 1:** Features of the patients enrolled in the present analysis.

Patient n.	Gender	Genotype (base change)	Age at diagnosis (years)	Age at HSCT (years)	Conditioning	Donor	Cell source	Infused CD34+ (10^6^/Kg)	Last chimerism recorded	Last IDUA recorded (nmol/h/mg- ref. 11–43)
1	M	c.152G > A hom	0.41	0.71	BU, CY	UNR	CB	8.9	100%	29.70
2	F	c.159C > A;c.653 T > C	0.61	1.15	BU, CY	UNR	BM	12.6	100%	52.40
3	F	c.1205G > A; c.1251delC	0.54	2.57	BU, CY	UNR	BM	5.5	100%	37.10
4	F	c.1163delC; c.152G > A	1.14	3.54	BU, CY, FLU	UNR	BM	7.6	95%	43.00
5	M	c.1205G > A; c.1738 + 5G > A	1.31	2.21	BU, CY	UNR	BM	8.9	70%	30.00
6	F	c.979G > C hom	1.51	2.54	BU, CY, FLU	UNR	BM	9	100%	43.50
7	F	c.208C > T; c.1487C > G	0.91	1.35	BU, CY	UNR	BM	11.9	100%	59.60
8	F	c.208C > T; c.1487C > G	1.24	2.80	BU, CY	UNR	BM	13.4	100%	46.90
9	M	c.1166_1171dup hom	1.46	1.77	BU, CY	REL	BM	19.3	100%	37.20
10	F	c.208C > T; c.1487C > G	0.69	1.02	BU, CY	UNR	CB	0.6	75%	32.70
11	F	c.208C > T; c.1487C > G	1.28	1.72	BU, CY	UNR	CB	0.3	100%	51.00
12	F	c.208C > T; c.398_403del6	0.70	1.05	BU, CY	UNR	CB	0.5	100%	52.20
13	F	c.1205G > A hom	0.69	0.94	BU, CY	UNR	CB	0.2	100%	68.00
14	F	c.1487C > G hom	0.66	0.98	BU, CY	UNR	CB	9.4	80%	24.80
15	M	c.1487C < G; c.1598C > G	1.18	1.65	BU, CY	REL	BM	15.5	95%	40.40

Patients 3,4 and 8 underwent two HSCT and the data are recorded with reference to the second transplantation.

Abbreviations: UNR: unrelated donor; REL: related donor; CB: cord blood; BM: bone marrow; BU: busulphan; CY: cyclophosphamide; FLU: fludarabine; IDUA: leucocyte alpha-L- iduronidase.

Patients 2, 4 and 6 received a matched unrelated bone marrow graft which was T-cell depleted by CD34+ cell enrichment. Patients 7 and 9 received an unmanipulated bone marrow graft from a matched unrelated and a matched non-carrier sibling donor respectively. Following the implementation by the European Blood and Marrow Transplantation (EBMT) group of international guidelines for HSCT in MPS patients in 2005, patients 1,10,11,12,13,14 have been transplanted with an unrelated cord blood unit. All patients in this series had a conditioning regimen including busulfan and cyclophosphamide (*plus* fludarabine in patients 4 and 6).

Nine out of 15 (60.0%) patients received ERT before and soon after HSCT. ERT was delivered for a median time of 0.47 years (5.6 months, range 3.2 to 9.0 months): on average, 0.28 years (3.4 months) before and 0.19 years (2.3 months) after HSCT.

Upon the latest hematological follow-up, leukocyte enzyme activity of all the 15 patients fell within normal values (median value: 43 nmol/h/mg; range: 24.8–68.0; reference range > 11.0). GAGs levels, remarkably high upon diagnosis (211 ± 203 mg/g creatinine), dropped within or just above the upper reference range for age (9.7 ± 6.8 ng/g creatinine, *p* < 0.001) by the ninth month after HSCT. Five patients show stable mixed chimerism and all the other recipients show a full donor chimera.

### 3.2 Growth during the first 5 years after HSCT

**Fig. 1:**
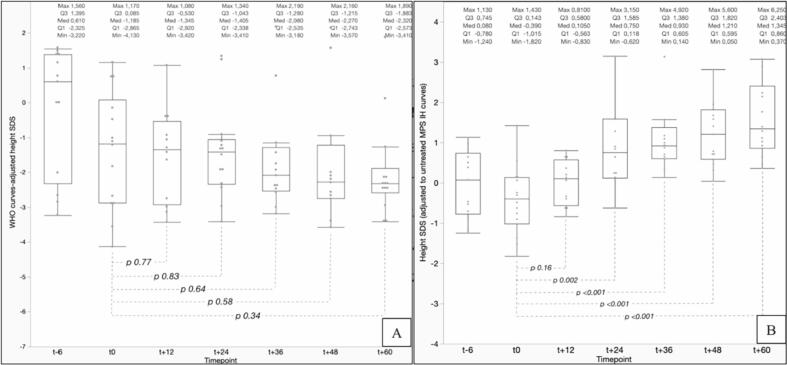
A. Box plots showing median and interquartile ranges for the height SDS values, recorded with reference to the general pediatric population (WHO charts), for the 15 patients enrolled, from 6 months before (t_−__6_) to 5 years after HSCT (t_60_). Despite a progressive height loss, height SDS at each timepoint is not statistically lower than stature recorded upon transplantation. B. Box plots showing median and interquartile ranges for the height SDS values, recorded with reference to charts of MPS-IH untreated patients, for the 15 children enrolled, from 6 months before (t_−__6_) to 5 years after HSCT (t_60_). Our data showed a progressive and statistically significant increase in height SDS.

The median value of height SDS for the patients enrolled, estimated with reference to the WHO growth charts, showed a progressive decrease during the first 5 years after HSCT, though the difference between t_0_ (−1.19 SDS; range: −4.13 to +1.17) and any subsequent timepoint (included t_60_, −2.32 SDS; range: −3.41 to +1.89) was not statistically significant (*p* 0.34 at t_60_, Fig. 1A).

Nevertheless, when comparing the stature recorded for each patient at each timepoint with the curves of untreated MPS-IH patients, median height SDS in our population of transplanted patients showed a progressive and statistically significant increase from t_0_ (−0.39 SDS; range: −1.82 to +1.43) to t_60_ (+1.35 SDS; range: +0.37 to +6.25, *p* < 0.001) (Fig. 1B). Median height SDS values were statistically higher than t_0_ from t_24_ onwards, with statistical significance progressively increasing in time.

**Fig. 2:**
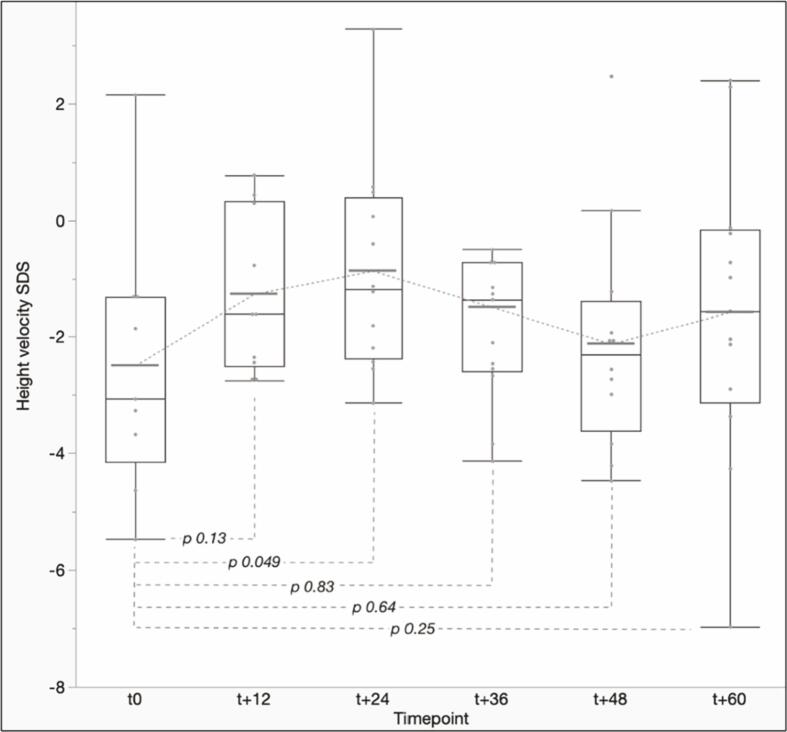
Box plots showing median and interquartile ranges for height velocity SDS, estimated with reference to the general pediatric population, recorded during the 5-years follow-up after HSCT. The solid horizontal line represents the mean values recorded at each timepoint.

As showed in Fig. 2, height velocity showed a remarkable acceleration after HSCT, with the greatest increase being recorded after 24 months (mean height velocity at t24: −0.84 ± 1.79 SDS *versus* − 2.49 ± 2.24 SDS at t_0_, *p* 0.049). Despite a subsequent reduction, recorded mean height velocity SDS was higher than t_0_ for all the remaining timepoints.

### 3.3 Growth at t_9yrs_ and at final height

Fourteen patients had achieved t_9yrs_, while 7 had attained final height.

**Fig. 3:**
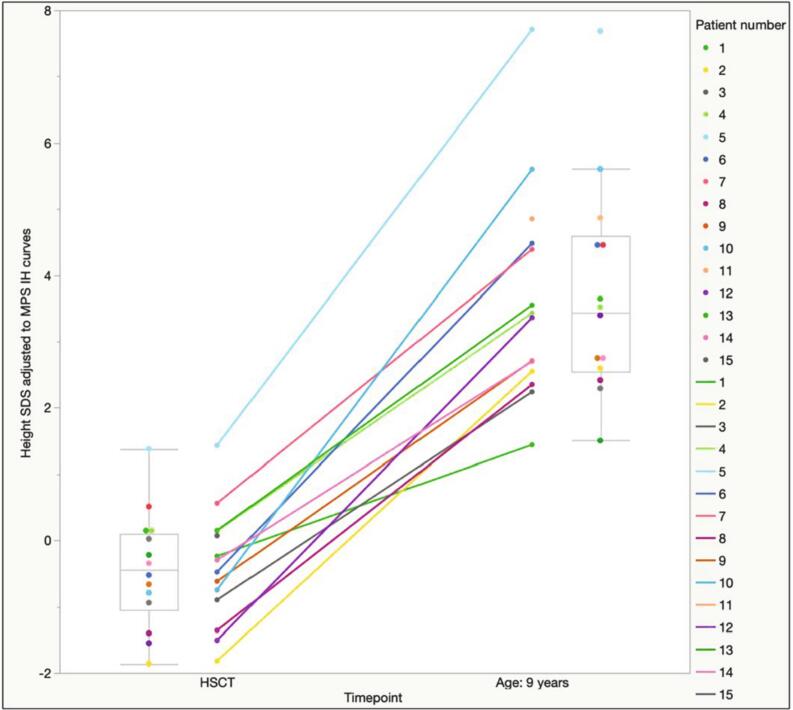
Patient-by-patient increase in height SDS from t0 to the age of 9 years. Height SDS are estimated with reference to the untreated MPS-IH-specific curves. Box plots for median and interquartile range are represented in light gray.

When estimating height SDS with reference to untreated MPS-IH patients, 14 out of 14 patients presented with a remarkable increase in height SDS from t_0_ to t_9yrs_ (mean height SDS +3,67 ± 1.63 SDS at t_9yrs_ *versus* − 0.4 ± 0.86 at t_0_, *p* < 0.0001 – height gain: 4.07 ± 1.27 SDS). Fig. 3 shows the patient-by-patient increase in height SDS with reference to untreated MPS-IH patients.

**Fig. 4:**
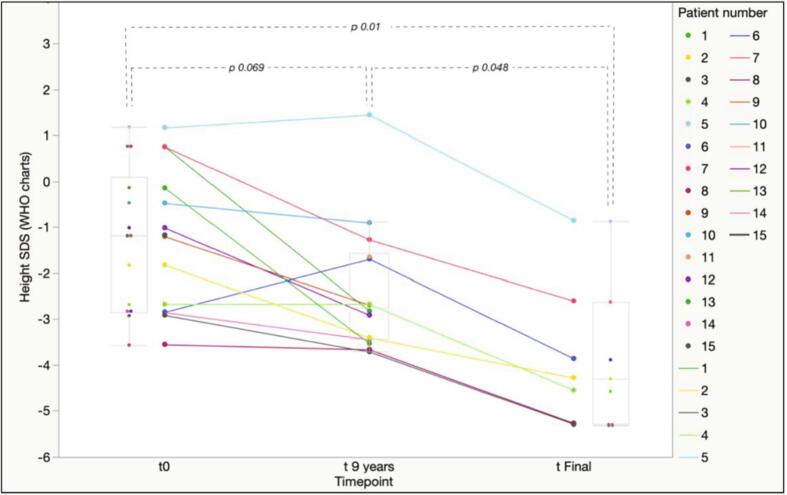
Patient-by-patient changes in height SDS at t_0_, t_9 years_ and T_final_ with reference to the WHO growth charts for the general pediatric population. Box plots for median and interquartile range for each timepoint are represented in light gray.

On the other hand, when referred to the WHO growth charts (Fig. 4), height SDS showed a progressive decrease from t_0_ to t_9yrs_ in 11 out of 14 patients (78.6%), while 3 showed either a stable or an increased height SDS at t_9yrs_. Accordingly, mean height SDS was lower at t_9yrs_ (−2.35 ± 1.43) than at t_0_ (−1.28 ± 1.55), though the difference was not statistically significant (*p* 0.069).

Six out of the 7 patients who attained final height presented with short stature (height < −2 SDS). The only patient with a normal final height was a male, 170.1 cm tall (−0.85 SDS, WHO charts), remarkably below his MPH (∆ MPH-H SDS = 2.14 SDS). Overall, all the adult patients presented with a mean final height (−3.81 ± 1.60 SDS) remarkably below their MPH (∆ MPH-H SDS = 3.61 ± 1.33 SDS) and statistically lower than the one recorded at t_9yrs_ (*p* 0.048) and at t_0_ (*p* 0.01).

**Fig. 5:**
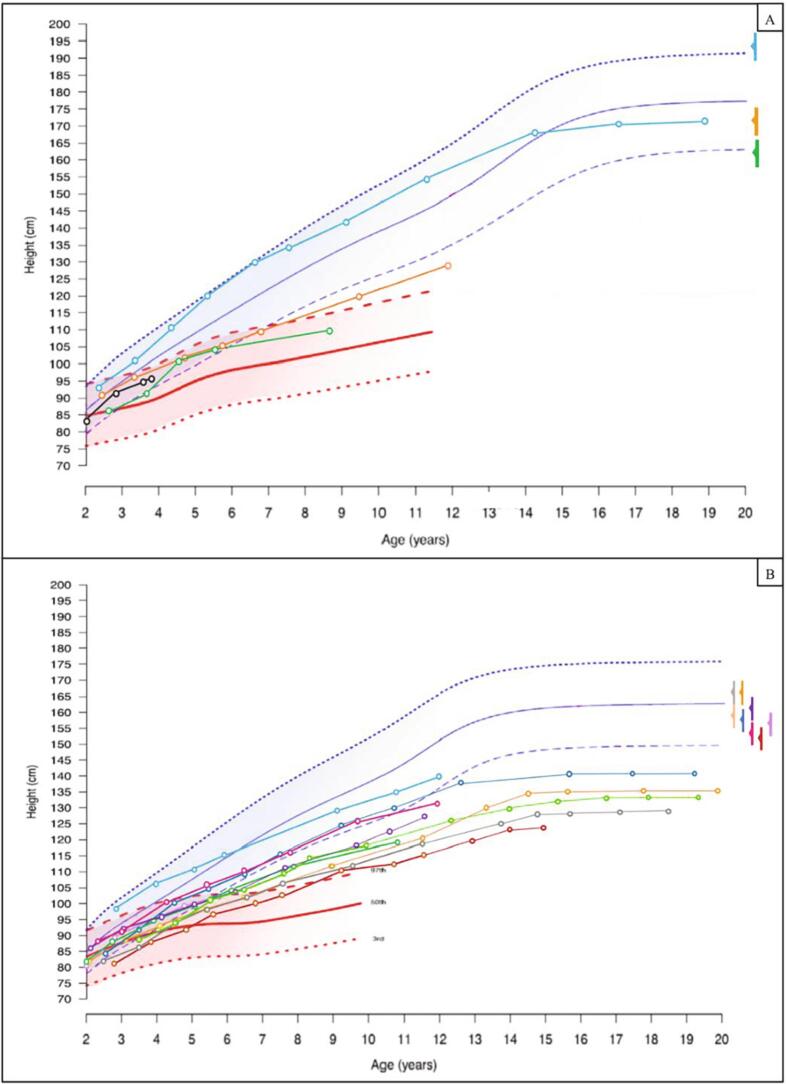
Growth patterns for the 15 patients enrolled, plotted against the WHO growth charts for the general pediatric population (blue-shadowed) and against the untreated MPS-IH growth charts (red-shadowed) in males (Fig. 5A) and females (5B). Each height value recorded is represented with a circle. Midparental height for each patient is represented with a color-matched arrow on the right of the charts, with a solid vertical line representing ± 1 SDS variability.

Fig. 5 shows the growth recorded for the patients enrolled, plotted against the WHO and MPS-IH-specific growth charts.

**Fig. 6:**
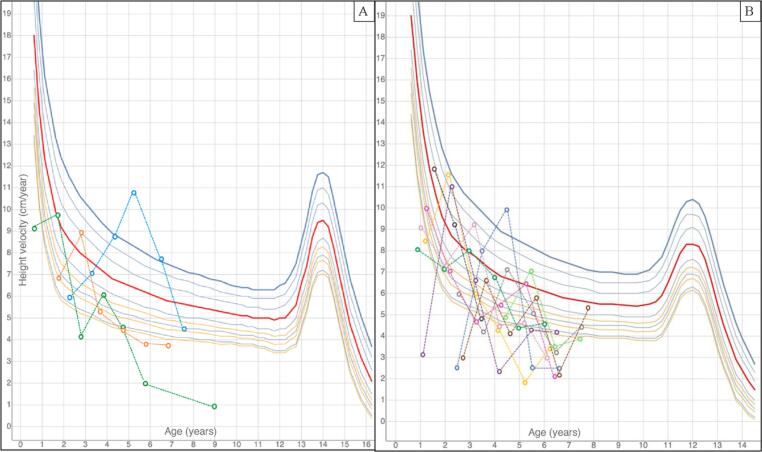
Height velocity (cm/year) for males (6A) and females (6B), plotted against the Tanner height velocity growth charts for the general pediatric population. Each height velocity value is represented with a circle and is plotted from t_0_ onwards (at t_0_, t_12_, t_24_, t_36_, t_48_ and t_60_). In most patients, transplantation (t_0_) was followed by a remarkable increase in height velocity, followed by a progressive decline with age.

Fig. 6 represents the height velocity, plotted against the Tanner growth chart for healthy patients, recorded at transplantation (t_0_) and subsequently yearly for the first 5 years after HSCT. A remarkable increase of height velocity was recorded at t_12_ and/or t_24_ in most patients.

### 3.4 Factors affecting growth

**Fig. 7:**
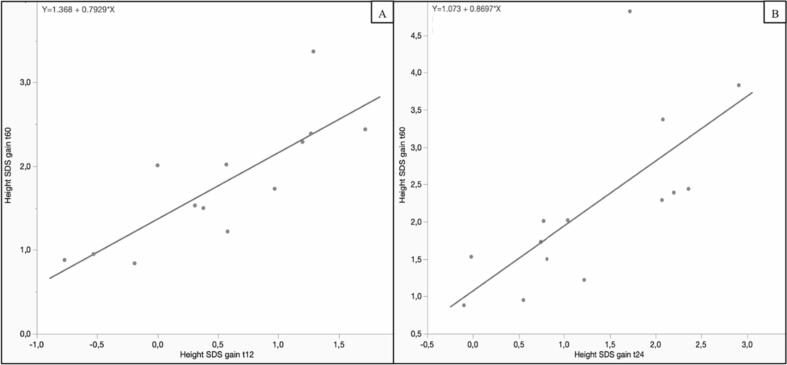
Linear regression model showing the relationship between height gain SDS at t_12_ (Fig. 7 A) and at t_24_ (7 B) on the X axis with the overall height SDS gain 60 months after HSCT on the Y axis.

On a linear regression model, we found that the height gain recorded at t_12_ (Fig. 7A) and t_24_ (7B) statistically correlated with the overall height gain at t_60_ (t_12_: R^2^ 0.66, Pearson's r 0.81, *p* 0.0013; t_24_: R^2^ 0.50, Pearson's r 0.70, *p* 0.007). The graphical and mathematical relationship is represented in Fig. 7.

However, on a multiple regression model, none among height gain at t_12_, age at HSCT, birth length, latest chimerism recorded or MPH were found to statistically affect the overall outcome at t_60_.

## 4 Discussion

HSCT has strikingly improved the overall clinical course and life expectancy in patients with MPS-IH. Accordingly, the dramatic increase in the survival rates of transplanted patients has led to the awareness that the detection and systematic assessment of several disease-related disorders need a long-term follow-up. Indeed, though it has been widely demonstrated that HSCT results in a remarkable reduction of the incidence and severity of several complications, a residual disease-related burden remains [22].

From an auxological perspective, the severe impairment of linear growth in untreated MPS-IH patients had been already assessed systematically in 2015 in a study held on 16 MPS-IH patients with Hurler syndrome [26]: these children presented with a regular growth (height between 0 and + 2 SDS) during the first 2 years of life, but subsequently showed a progressive drop of height SDS, resulting in severe short stature. More recently, Viskochil and colleagues have published the MPS-IH-specific growth charts for untreated patients [24].

On the other hand, only few papers have systematically assessed the potential height gain provided by HSCT and all the figures published so far merely provide a comparison with the healthy population of gender- and age-matched children.

In a wide study assessing the outcomes of transplantation on a population of children transplanted for Hurler syndrome, Aldenhoven and colleagues briefly described linear growth as a progressive decrease in height centiles, with most of the deceleration occurring after the age of 10 years [22]. This trend was described regardless of the conditioning schedule administered before HSCT, as the study population included both TBI- and chemo only-conditioned children. Given the described but not fully predictable detrimental effect of TBI on growth, we excluded irradiated patients, in order to systematically assess the independent contribution of transplantation.

In three additional papers, the Authors describe a somehow regular growth during the first years after HSCT, followed by a progressive decrease in height velocity, with height SDS dropping below −2 SDS by the sixth year after transplantation [17,23,27].

When the auxological data recorded are plotted against the WHO charts, our findings are superimposable with those previously published, with 9 out of 15 patients being shorter than −2 SDS at t_9yrs_. Among 7 patients who had attained their final height, one presented with a normal height, one was shorter than −2 SDS while the remaining 5 were shorter than −3 SDS, with their mean final height falling 3.81 ± 1.60 SDS below the median stature for the general population and 3.61 ± 1.33 SDS below their parental growth potential.

Overall, growth velocity was less severely affected during the first 5 years after HSCT and, in general, up to the ninth year of life, while it showed a more remarkable impairment subsequently. This might be at least partially regarded as the consequence of a severely attenuated growth spurt experienced during puberty, as already demonstrated for different types of mucopolysaccharidoses [[28], [29], [30]]. During this crucial phase, the synergic action of growth hormone and sexual hormones, key elements for the pubertal increase in height velocity in adolescence, may be variably hampered due to the pathological storage of GAGs in the growth plates and the gap between affected and healthy patients becomes remarkably overt.

Overall, our data confirm that HSCT is not effective in restoring the full growth potential when comparing patients with MPS-IH to healthy individuals. Nevertheless, the recent publication of the growth charts for untreated MPS-IH patients allowed us to perform, for the first time, a systematic comparison between transplanted and untreated individuals.

Our data showed that patients experienced a progressive and statistically significant improvement in height SDS after HSCT, when compared to transplantation-naïve MPS-IH individuals. For all the patients enrolled, the mean height gain recorded by the ninth year of life, compared to untreated MPS I—H, was greater than 4 SDS. Thus, by restoring alpha-L-iduronidase (IDUA) activity, we hereby demonstrate that HSCT plays a remarkable positive role on growth, compared with untreated patients, though its beneficial effects seem poor when assessed with reference to the unaffected pediatric population. This confirms that part of the burden of disease involving the bones and growth plates could not be reverted by the reestablished IDUA activity provided by HSCT.

Height velocity improved mostly by the end of the second year after transplantation, when it became statistically greater than the baseline. The latency between transplantation and the peak of growth acceleration recorded may find several explanations: firstly, patients usually experience a reduction in height velocity for the first months after transplantation, as a result of a combination of detrimental factors (poor feeding, malabsorption in case of graft *versus* host disease, steroids to treat HSCT-related complications, infectious complications); secondly, the IDUA activity takes several weeks before being fully restored, also in relation to the time of engraftment.

The systematic estimation of height SDS with reference to the curves of untreated MPS-IH represents the main source of novelty of our analysis. In addition, the homogeneity of the sample (in terms of age, therapeutic approach and conditioning schedule undertaken, with exclusion of irradiated patients) allowed us to avoid pre-analytical *biases*.

On the other hand, we are aware of some limitations. Firstly, as no standardized growth data are available for treatment-naïve MPS-IH patients older than 10 years, mostly due to the unfavorable prognosis of the untreated pathology, we could not draw conclusions on the overall height gain in treated *versus* untreated patients upon final height attainment. Nevertheless, height SDS showed a progressive increase, when plotted against untreated patients, with height SDS gain at t_9yrs_ being greater than any other recorded earlier, *i.e.* during the first 5 years after transplantation. These figures suggest that height gain was a progressive phenomenon and we may hypothesize that it continues also after t_9yrs_. It may be argued that, when comparing our patients to healthy children, most of the reduction in terms of final height SDS occurred during adolescence. However, as previously discussed, it is likely that most of the overall height SDS loss experienced by our patients was due to an attenuated pubertal spurt. When comparing treated *versus* untreated patients, it is very unlikely that the pubertal spurt hypothetically experienced by the latter could be greater than that of transplanted individuals. As most untreated individuals with MPS-IH die within 10 years of life, this hypothesis cannot be confirmed in the same disease, but it is well known that a very reduced pubertal spurt has been demonstrated in other untreated MPSs. [22]. Overall, we believe that it is extremely unlikely that the height gain recorded by t9_yrs_ could be lost later, in comparison to untreated patients.

Secondly, the sample size was overall small, though noteworthy when considering the rarity of the disease and the strict criteria that candidate MPS-IH patient to transplantation.

Lastly, it could be objected that our comparison of few transplanted individuals with the growth chart of untreated MPS-IH is weakened by the genotype and phenotype heterogeneity of the disease; nevertheless, it is known that phenotype heterogeneity is extremely limited in the severe forms of lysosomal storage disorders (as in our patients), in contrast with the attenuated forms [31].

In conclusion, though not efficient enough to restore a normal growth pattern in MPS-IH patients, we hereby demonstrate that hematopoietic stem cell transplantation positively affects growth and provides transplanted patients with a remarkable height gain compared to untreated gender- and age- matched individuals.

## Declaration of Competing Interest

RP has received travel grants and honoraria for speaking engagements from Sanofi-Genzyme.

## References

[1] Neufeld E., Muenzer J., Scriver C.R., Beaudet A.L., Sly W.S., Valle D. (2001). The mucopolysaccharidoses. The Metabolic & Molecular Bases of Inherited Disease [Internet]. III.

[2] Simonaro C.M., D’Angelo M., He X., Eliyahu E., Shtraizent N., Haskins M.E. (2008 Jan). Mechanism of glycosaminoglycan-mediated bone and joint disease. Am. J. Pathol. [Internet]..

[3] Kollmann K., Pestka J.M., Kühn S.C., Schöne E., Schweizer M., Karkmann K. (2013 Dec). Decreased bone formation and increased osteoclastogenesis cause bone loss in mucolipidosis II. EMBO Mol. Med..

[4] Gardner C.J., Robinson N., Meadows T., Wynn R., Will A., Mercer J. (2011 Apr 21). Growth, final height and endocrine sequelae in a UK population of patients with Hurler syndrome (MPS1H). J. Inherit. Metab. Dis. [Internet].

[5] Melbouci M., Mason R.W., Suzuki Y., Fukao T., Orii T., Tomatsu S. (2018 May). Growth impairment in mucopolysaccharidoses. Mol. Genet. Metab..

[6] Patel P., Suzuki Y., Tanaka A., Yabe H., Kato S., Shimada T. (2014). Impact of enzyme replacement therapy and hematopoietic stem cell therapy on growth in patients with Hunter syndrome. Mol. Genet. Metab. Rep. [Internet].

[7] Noh H., Lee J.I. (2014 Jun). Current and potential therapeutic strategies for mucopolysaccharidoses. J. Clin. Pharm. Ther. [Internet].

[8] Wraith J.E. (1995 Mar 1). The mucopolysaccharidoses: a clinical review and guide to management. Arch. Dis. Child [Internet].

[9] Taylor M., Khan S., Stapleton M., Wang J., Chen J., Wynn R. (2019 Jul). Hematopoietic stem cell transplantation for mucopolysaccharidoses: past, present, and future. Biol. Blood Marrow Transplant. [Internet]..

[10] Guffon N., Bertrand Y., Forest I., Fouilhoux A., Froissart R. (2009 May). Bone marrow transplantation in children with hunter syndrome: outcome after 7 to 17 years. J. Pediatr. [Internet]..

[11] Wraith J.E., Beck M., Lane R., van der Ploeg A., Shapiro E., Xue Y. (2007 Jul). Enzyme replacement therapy in patients who have mucopolysaccharidosis I and are younger than 5 years: results of a multinational study of recombinant human alpha-L-iduronidase (laronidase). Pediatrics..

[12] Schulze-Frenking G., Jones S.A., Roberts J., Beck M., Wraith J.E. (2011 Feb). Effects of enzyme replacement therapy on growth in patients with mucopolysaccharidosis type II. J. Inherit. Metab. Dis..

[13] Doherty C., Stapleton M., Piechnik M., Mason R.W., Mackenzie W.G., Yamaguchi S. (2019 Jul). Effect of enzyme replacement therapy on the growth of patients with Morquio A. J. Hum. Genet..

[14] Harmatz P., Hendriksz C.J., Lampe C., McGill J.J., Parini R., Leão-Teles E. (2017). The effect of galsulfase enzyme replacement therapy on the growth of patients with mucopolysaccharidosis VI (Maroteaux-Lamy syndrome). Mol. Genet. Metab. [Internet].

[15] Yabe H., Tanaka A., Chinen Y., Kato S., Sawamoto K., Yasuda E. (2016 Feb). Hematopoietic stem cell transplantation for Morquio A syndrome. Mol. Genet. Metab. [Internet]..

[16] Chinen Y., Higa T., Tomatsu S., Suzuki Y., Orii T., Hyakuna N. (2014). Long-term therapeutic efficacy of allogenic bone marrow transplantation in a patient with mucopolysaccharidosis IVA. Mol. Genet. Metab. Rep. [Internet]..

[17] Guffon N., Souillet G., Maire I., Straczek J., Guibaud P. (1998 Jul). Follow-up of nine patients with hurler syndrome after bone marrow transplantation. J. Pediatr. [Internet]..

[18] Jiang Z., Byers S., Casal M.L., Smith L.J. (2020 Dec 16). Failures of endochondral ossification in the mucopolysaccharidoses. Curr. Osteoporos. Rep. [Internet].

[19] Tomatsu S., Alméciga-Díaz C.J., Montaño A.M., Yabe H., Tanaka A., Dung V.C. (2015 Feb). Therapies for the bone in mucopolysaccharidoses. Mol. Genet. Metab. [Internet]..

[20] Muenzer J., Wraith J.E., Clarke L.A. (2009 Jan 1). Mucopolysaccharidosis I: management and treatment guidelines. Pediatr. Int..

[21] Staba S.L., Escolar M.L., Poe M., Kim Y., Martin P.L., Szabolcs P. (2004). Cord-blood transplants from unrelated donors in patients with Hurler’s syndrome. N. Engl. J. Med..

[22] Aldenhoven M., Wynn R.F., Orchard P.J., O’Meara A., Veys P., Fischer A. (2015 Mar 26). Long-term outcome of Hurler syndrome patients after hematopoietic cell transplantation: an international multicenter study. Blood [Internet].

[23] Souillet G., Guffon N., Maire I., Pujol M., Taylor P., Sevin F. (2003). Outcome of 27 patients with Hurler’s syndrome transplanted from either related or unrelated haematopoietic stem cell sources. Bone Marrow Transplant..

[24] Viskochil D., Clarke L.A., Bay L., Keenan H., Muenzer J., Guffon N. (2019 Dec 22). Growth patterns for untreated individuals with MPS I: Report from the international MPS I registry. Am. J. Med. Genet. A [Internet].

[25] Tanner J.M., Whitehouse R.H. (1976 Mar). Clinical longitudinal standards for height, weight, height velocity, weight velocity, and stages of puberty. Arch. Dis. Child [Internet]..

[26] Różdżyńska-Świątkowska A., Jurecka A., Cieślik J., Tylki-Szymańska A. (2015 Aug 20). Growth patterns in children with mucopolysaccharidosis I and II. World J. Pediatr. [Internet].

[27] Aldenhoven M., Boelens J.J., de Koning T.J. (2008). The clinical outcome of hurler syndrome after stem cell transplantation. Biol. Blood Marrow Transplant..

[28] Rozdzynska A., Tylki-Szymanska A., Jurecka A., Cieslik J. (2011). Growth pattern and growth prediction of body height in children with mucopolysaccharidosis type II. Acta Paediatr. Int. J. Paediatr..

[29] Quartel A., Hendriksz C.J., Parini R., Graham S., Lin P., Harmatz P. (2015). Growth charts for Individuals with mucopolysaccharidosis VI (Maroteaux-Lamy Syndrome). JIMD Rep. [Internet].

[30] Parini R., Jones S.A., Harmatz P.R., Giugliani R., Mendelsohn N.J. (2016 Apr). The natural history of growth in patients with Hunter syndrome: data from the Hunter Outcome Survey (HOS). Mol. Genet. Metab. [Internet]..

[31] Gieselmann V. (2007 Jan 2). What can cell biology tell us about heterogeneity in lysosomal storage diseases?. Acta Paediatr [Internet].

